# Characteristics of the most severely ill and injured patients in a Norwegian helicopter emergency medical service: a retrospective cohort study

**DOI:** 10.1186/s12873-024-00954-7

**Published:** 2024-03-02

**Authors:** Eirik Ringen, Helge Haugland, Jostein Rødseth Brede

**Affiliations:** 1 Faculty of Medicine and Health Sciences, Norwegian University of Science and Technology, St. Olavs Hospital, Trondheim, Norway; 2 Department of Emergency Medicine and Pre-hospital Services, St. Olavs University Hospital, Trondheim, Norway; 3 Department of Research and Development, Norwegian Air Ambulance Foundation, Oslo, Norway; 4 Department of Anesthesiology and Intensive Care Medicine, St. Olavs University Hospital, Trondheim, Norway

**Keywords:** Helicopter emergency medical service (HEMS), Return of spontaneous circulation (ROSC), National committee for aeronautics (NACA), Pre hospital interventions, Critically ill

## Abstract

**Background:**

Physician-staffed helicopter emergency medical services (HEMS) are dispatched to a variety of incidents, ranging from less serious to life-threatening. The skillset of a physician may be important to provide appropriate care for the most critically ill and severely injured patients. A better understanding of these patients may therefore be important to optimize dispatch criteria, training, and equipment setups for HEMS units.

The aim of this study was to describe the characteristics of patients with the national advisory committee on aeronautics (NACA) score 5 and 6, primarily by diagnostic group and interventions performed.

**Methods:**

Retrospective cohort study on aggregated data from the HEMS-base in Trondheim, Norway. All patients with NACA score 5 and 6 in the 10-year period from 2013 to 2022 were included. Patients with return of spontaneous circulation (ROSC) after successful cardiopulmonary resuscitation were described separately from non-cardiac arrest patients.

**Results:**

Out of 9546 patient encounters, 2598 patients were included, with 1640 in the NACA 5 and 958 in NACA 6 group. Patient age was median 63 (interquartile range 45–74) and 64% of the patients were male. Post-ROSC patients accounted for 24% of patients. Of the non-cardiac arrest patients, the most frequent aetiology was trauma (16%), cardiac (15%), neurologic (14%) and respiratory (11%). The most common physician-requiring advanced interventions were general anaesthesia (22%), intubation (21%), invasive blood pressure monitoring (21%) and ventilator treatment (18%). The mean number of advanced interventions per mission were consistent during the study period (1,78, SD 0,25).

**Conclusion:**

Twenty-seven percent of all HEMS dispatches were to NACA 5 and 6 patients. Twenty-four percent of these were post-ROSC patients. Sixty-three percent of all patients received at least one advanced physician-requiring intervention and the average number of interventions were consistent during the last 10 years. Hence, the competence a physician-staffed HEMS resource provide is utilized in a high number of critically ill and injured patients.

**Supplementary Information:**

The online version contains supplementary material available at 10.1186/s12873-024-00954-7.

## Background

The Norwegian nationwide physician-staffed helicopter emergency medical service (HEMS) consists of 13 helicopter bases and seven fixed wing bases, in addition to seven search and rescue helicopter bases commissioned by the Royal Norwegian Air Force and CHC Helicopter service [1, 2]. These HEMS bases cover the Norwegian mainland and the coastal areas.

The dispatch of HEMS units is coordinated by the regional emergency medical communication centre (EMCC) [3]. Illness and incident severity are assessed by the EMCC operator, usually a trained nurse or paramedic. Assessment is based on criteria described in “Norwegian Index of Medical Emergencies” [4]. A dedicated HEMS-coordinator assess the necessity of HEMS dispatch for all calls received by the EMCC [3], based on need for additional competence, time criticality and/or accessibility to the patient. This assessment is not based upon objective criteria, but guidelines utilized by the HEMS EMCC coordinator (Supplementary file 1). Objective dispatch criteria and dispatch precision is currently being investigated [5, 6]. Ultimately the HEMS physician decide whether or not to respond to a dispatch request. After a mission is completed, the severity of the disease or injury is graded by the HEMS physician, according to the national advisory committee on aeronautics (NACA) score [7, 8] (Table 1).

**Table 1:** The National Advisory Committee on Aeronautics (NACA) score [8]

Score	Description
NACA 0	No injury or disease
NACA 1	Injuries/diseases without any ned for acute physician’s care
NACA 2	Injuries/diseases requiring examination and therapy by a physician, but hospital admission is not indicated
NACA 3	Injuries/diseases without acute threat to life but requiring hospital admission
NACA 4	Injuries/diseases that can possibly lead to deterioration of vital signs
NACA 5	Injuries/diseases with acute threat to life
NACA 6	Injuries/diseases transported after successful resuscitation of vital signs
NACA 7	Lethal injuries or diseases (with or without resuscitation attempts)

Patients who die during the HEMS mission are graded NACA 7. Hence, the NACA 5 and 6 groups are the most severely ill and injured patients treated by HEMS that survive. Treatment of this patient cohort may be challenging, as they often need advanced medical treatment in a challenging pre-hospital environment with limited access to equipment and personnel.

An increased understanding of these patients may contribute to improved accuracy in training, equipment setups and dispatch priorities for pre-hospital medical resources. The aim of this study is therefore to describe the characteristics of the most severely ill and injured patients, primarily by diagnostic group and interventions performed in the pre-hospital setting.

## Methods

### Design and study participants

This is a retrospective population-based cohort study on aggregated patient data. We included all patients with NACA score 5 and 6 in the 10-year period from January 1st, 2013, to December 31st, 2022. The NACA 5 and 6 scores are only utilized to identify the most critically ill and injured, and the scores are not subject to comparison.

Data were collected from an established data warehouse that automatically collects data entries from the HEMS journaling system LABAS (Normann IT, Trondheim, Norway). The data is coupled with data from EMCC. As NACA 6 is given after successful resuscitation, the study population consists of a large number of patients with cardiac arrest. These patients receive treatment according to cardiopulmonary resuscitation (CPR) guidelines [9, 10]. Hence, patients achieving return of spontaneous circulation (ROSC) after CPR receive similar interventions regardless of the underlying diagnose. This gives these patients an intervention profile that is affected by CPR protocol, as opposed to the non-CPR patients – where interventions are decided by the physician alone.

Due to this we described the total population in two separate cohorts – the ROSC-group and non-CPR group.

### Setting

The study was performed in the service area for the HEMS base in Trondheim, Norway. This base covers a mixed rural/urban catchment population of approximately 700,000 inhabitants [11]. The crew is dispatched in a helicopter, or in a rapid response car if the patient is close to the HEMS base or if weather conditions restrict flying. Norwegian HEMS is a 24/7/365 service, staffed with a consultant anaesthesiologist, a HEMS crew member (paramedic or nurse) and a pilot [2]. HEMS operate as a highly specialized capability, supplementing basic emergency medical services (EMS) in the area, e.g. ground ambulances and rural on-call physicians. EMS routinely provide emergency medical interventions, i.e. supplemental oxygen, analgesics, assisted ventilation, intravenous access. Interventions not available from basic EMS include endotracheal intubation, administration of vasoactive medication, general anaesthesia, arterial lines, use of ultrasound, central venous catheter, blood transfusion, neonatal incubator and/or thoracostomy. These interventions require the presence of a HEMS physician and is defined as “advanced intervention” in this study. This corresponds with previous descriptions of advanced HEMS interventions [12].

The missions of Norwegian HEMS are defined as “primary” or “secondary”, secondary being inter-hospital transfers.

### Data variables

We extracted data such as patient age, sex, NACA score, diagnostic group and interventions performed. The diagnostic groups are derived from ICD-10 codes assigned by the HEMS physician. Each patient is assigned a primary diagnostic code based on the current clinical situation.

## Results

**Fig. 1:**
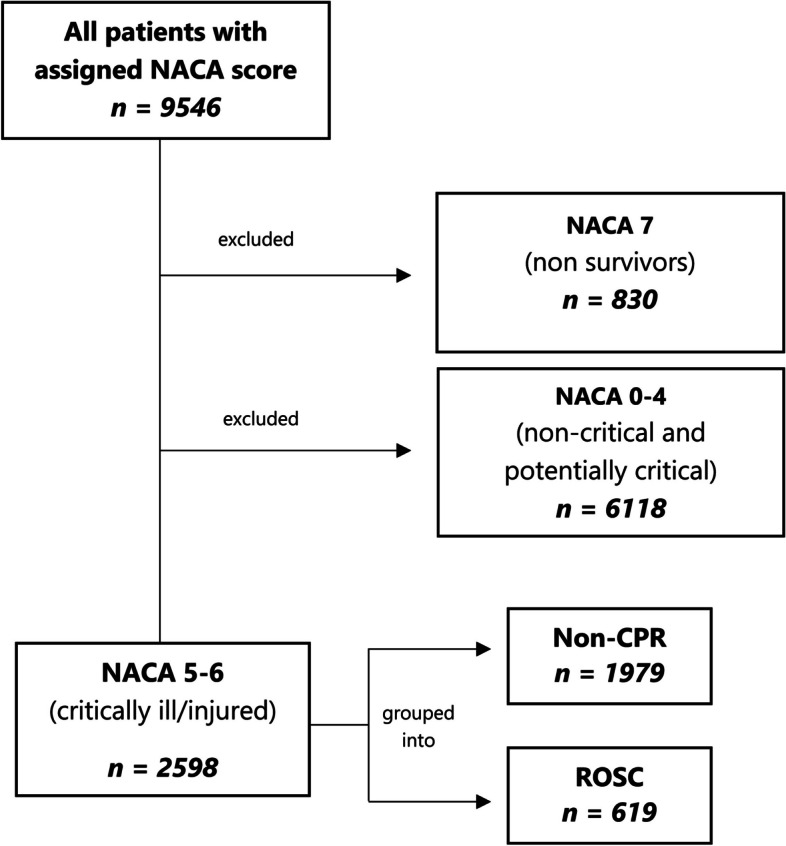
Flowchart of patient inclusion. CPR indicates “cardio-pulmonary circulation”, ROSC “return of spontaneous circulation”

In total, 9598 patients were assigned a NACA score in the study period, out of which 2598 were given score 5 (*n* = 1640) or 6 (*n* = 958) and therefore included in the study (Fig. 1). Hence, 27% of all patients retrieved by HEMS in the 10-year period was defined as severely ill or injured.

**Fig. 2:**
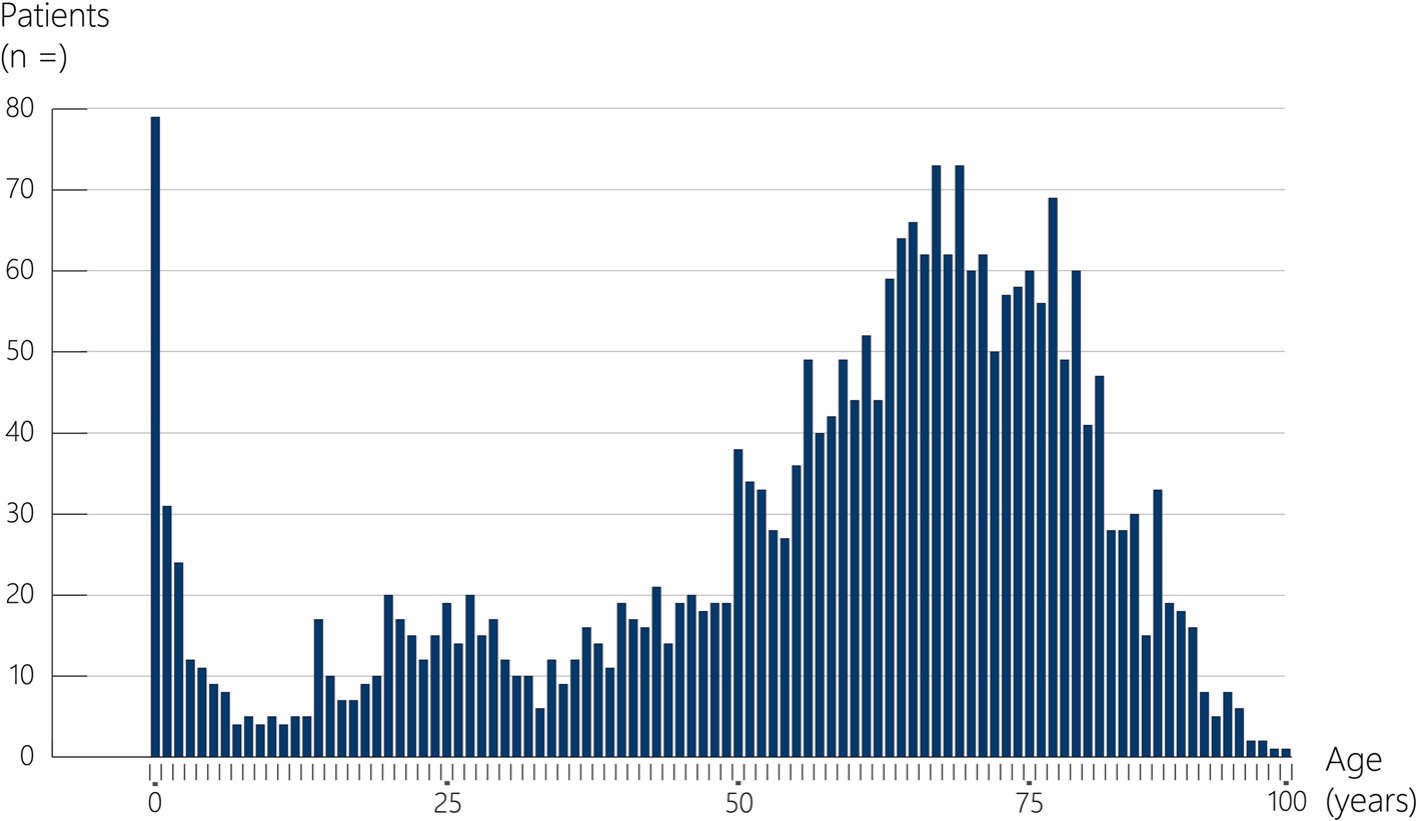
Age distribution

**Fig. 3:**
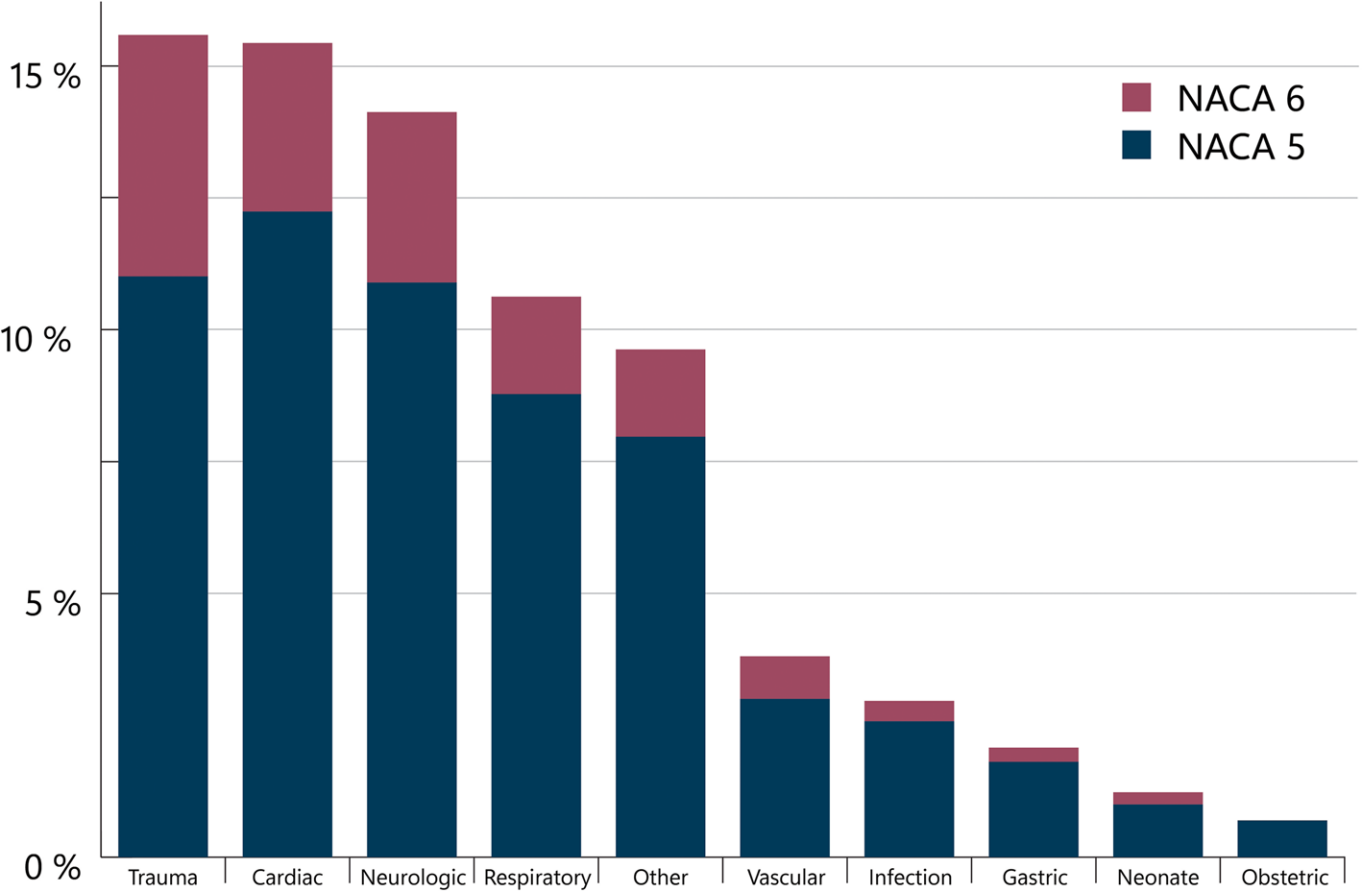
Distribution diagnostic groups in total NACA 5 and 6 patient population

Most patients were male (64%). Eighty percent of the patients were between 18 and 80 years, and 10% of patients older than 80 years. The paediatric population counts for 9%, with 3% being less than 1 year old (Fig. 2). Most patients in both ROSC and non-CPR group were found in the cardiac (35%), trauma (18%) and neurologic (15%) diagnostic groups. In the non-CPR group, the most prominent diagnostic groups were the trauma (16%), cardiac (15%) and neurologic (14%) (Fig. 3).

Across both groups, basic pre-hospital emergency interventions were performed in a high number of patients. The most frequent interventions within all diagnostic groups were administration of supplemental oxygen (77%), crystalloid/colloid infusion (63%), analgesics (34%) or other medications (44%).

**Fig. 4:**
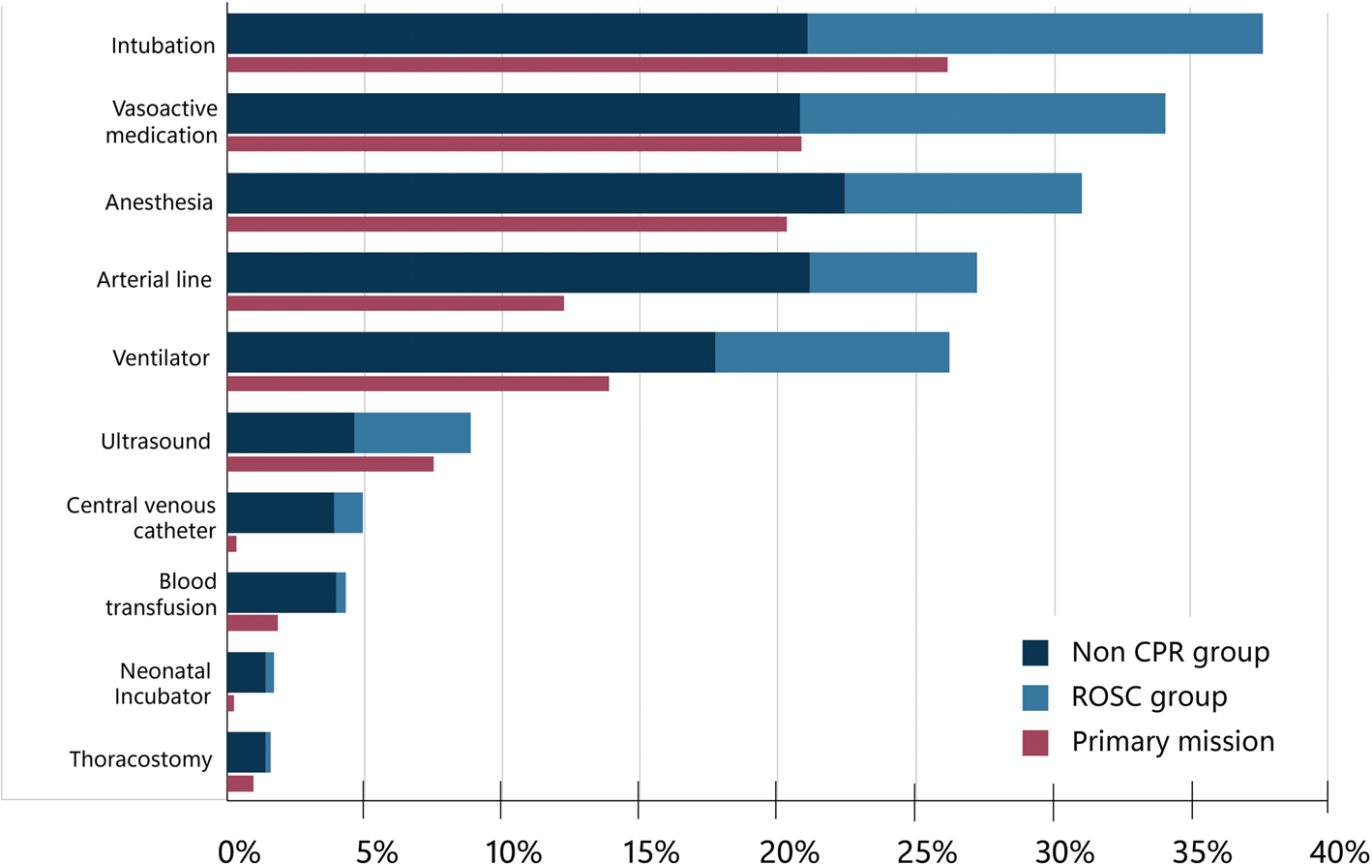
Advanced interventions performed in Non-CPR group and ROSC group, across total NACA 5 and 6 population

The most frequent advanced interventions were intubation (38%), anaesthesia (31%), invasive blood pressure monitoring (27%) and ventilator treatment (26%). Interventions such as blood transfusion (4%) or thoracostomy (1.6%) were rare (Fig. 4).

In total 93% of patients received an intervention of any type, while 61% received an advanced intervention. The average number of advanced interventions performed per patient every year were consistent (1,78, SD 0,25) with median 2,02 (IQR 1,65-1,95).

Primary missions accounted for 74% of the total missions. Figure 4 illustrates interventions performed in a primary or secondary mission.

Rapid response car was utilized in 29% of the cases. Cardiac arrests responded to by car accounted for 10% of the cases and 41% of the total cardiac arrest incidents.

A comprehensive table of patient data is available as supplementary material (Supplementary file 2).

## Discussion

Out of 9598 patients in the study period, 2598 patients (27%) were scored as NACA 5 and 6. These numbers are comparable to other Scandinavian studies, where 30% of the patients were scored as NACA 5 or 6 in a single-centre Swedish study, 20% in a national Danish study [13, 14], and 22% in a Norwegian study [12].

The non-CPR patients account for 76% of the study population, while 24% were post-ROSC patients. This is in concordance with the findings in a Danish study conducted by Alstrup et al., however this study also included the NACA 7 patients [13].

In the ROSC group the interventions reflect that treatment was done by CPR protocol. Cardiac related diagnostic codes were registered in 82% of these cases, which indicate underlying cardiac disease, the most common aetiology of cardiac arrest [15].

Among the advanced interventions in the non-CPR group, anaesthesia (22%), arterial line (21%), intubation (21%), and ventilator treatment (18%) were most common. These are all intensive care interventions. These patients are not treated according to a strict protocol, and the decision to perform intensive care interventions are based on the clinical findings in each patient. These interventions may not be possible without physician at scene. As these patients are severely ill or injured, it is fair to assume that these interventions were performed due to a potential benefit for the patient. However, our dataset does not consider vital parameters or other clinical information, thus it is difficult to establish whether 18–22% is a high, low or “correct” fraction of advanced interventions. Prospective studies, or retrospective studies with these data parameters registered, may answer this question. Further, the number of advanced treatments performed immediately after admission to hospital may provide an indication on potentially improvements in the pre-hospital intensive care interventions. However, this information was not available in our dataset.

Blood transfusion capability was first introduced to our system in 2016 [16]. Sunde et al. reported blood transfusions in 1.4% of responses in a five-year study at another Norwegian HEMS base [16]. Our study reports transfusion in 5% of NACA 5 and 6 patients. However, out of the total HEMS population 1.8% (169 of 9546) received transfusions, in concordance with Sunde et al. Hence, 66% of all pre-hospital blood transfusions is performed in the NACA 5 and 6 cohort (112 out of 169).

Invasive blood pressure monitoring in the ROSC group was performed in 26%. This may be a low number, considered that these patients are intensive care patients, treated by an anaesthesiologist and recommended post-ROSC treatment includes invasive blood pressure measurement [17]. However, factors such as short distance to hospital may influence this number and in 41% of the cardiac arrest calls HEMS dispatched in the rapid response car, which indicates close proximity to the hospital.

A small fraction of the total population were paediatric patients (9%), with 3% being less than 1 year and 38 neonates. Four of these were in cardiac arrest. Norwegian HEMS regularly transport neonates and infants in incubators between hospitals [2]. These missions count for most of the 3 % of patients < 1 year (52 entries of incubator use). These patients require intensive and specialized care, which is also reflected in the interventions registered for these missions. The low number of missions emphasize the importance of extensive training on conditions and contingencies that may occur in this patient group.

**Fig. 5:**
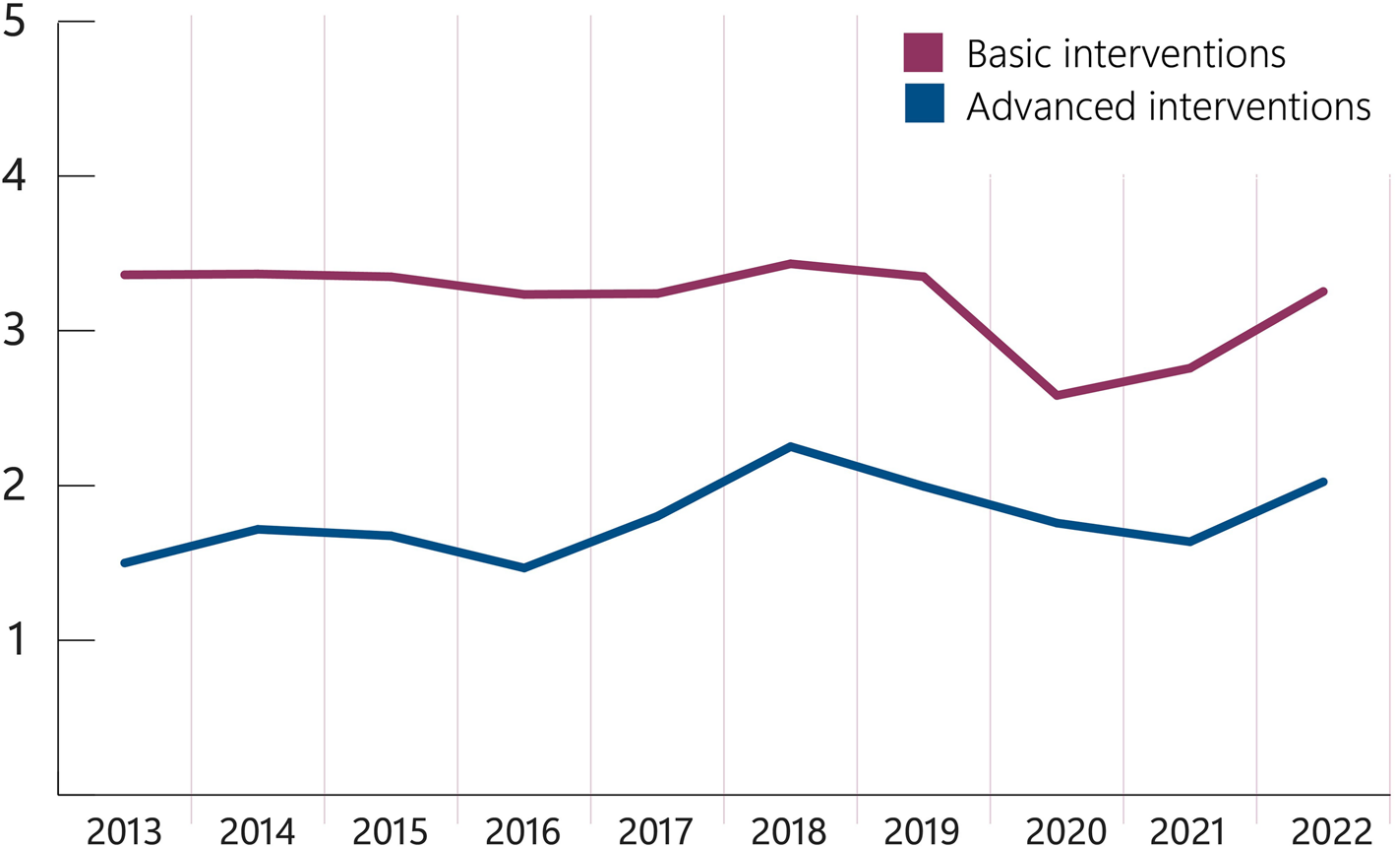
Advanced and basic interventions per patient in total NACA 5 and 6 population

Interventions such as ultrasound, blood transfusion, chest compression device and intra-osseous access were all implemented in HEMS during the study period. With increased possibilities in both advanced and basic interventions, an increase in interventions could be anticipated. However, during the study period the number of interventions registered per patient were consistent (Fig. 5). The COVID-19 pandemic may have influenced the number of interventions, with 2020 as the year with fewest interventions per patient. However, it is important to note that a decision to abstain from an intervention is an equally active decision as performing it [18], an option that is possible due to physician involvement. This cannot be quantified from the dataset; however, we assume this has effect on the number of interventions to any given patient.

Twenty-six percent were secondary missions, with patients admitted to a hospital prior to HEMS involvement. This may explain variations in advanced interventions performed in primary or secondary missions. Arterial lines and ventilator treatment were provided in a high number of secondary missions, as expected, since these are common hospital interventions in severely ill or injured patients. Neonatal inter-hospital transfers account for most of the incubator use. In secondary missions, it is less important where the advanced interventions where performed, however the follow-up still require the presence of an anaesthesiologist. Still, it is interesting that the majority of intubations (69%), anaesthesia (65%), vasoactive medication 61%) and ventilator treatment (55%) is performed in primary missions.

Both the dispatch process and accessibility to the patient by the HEMS crew may influence the distribution of NACA 5 vs NACA 6 patients. Local EMS can request support from HEMS or the EMCC can request immediate dispatch based on medical criteria. This affects the NACA distribution, with 48% NACA 6 in car responses vs 32% NACA 6 in helicopter responses.

### Strengths and limitations

The major strength of this study is its 10-year span, and inclusion of all patients in the NACA 5 and 6 groups. Data has been collected directly after HEMS mission completion, by a small number of physicians from one HEMS base, limiting information bias from collection or recording data [19, 20]. The NACA score is widely used in HEMS services all over the world, and has been validated as a tool to predict mortality [7]. Hence, we consider the data quality to be robust.

This study has limitations, the first is that it is a single centre study. Second, data is registered by the on-scene physician to the digital LABAS database upon completion of every mission. This may lead to loss of information or inaccuracies. Third, the pre-hospital setting is challenging, and limited information, time and diagnostic tools make precise assignments and correct diagnostic codes difficult. This may result in missing or erroneous registered data. The diagnoses are set by the HEMS physician based on the information available during the mission. Thus, these diagnoses may differ from diagnoses upon hospital discharge. Fourth, NACA score is set without strict objective criteria. This can result in selection bias because some patients may be registered as NACA 4 by individual physicians and NACA 5 by others. However, since this study describes NACA 5 and 6, and the criteria between NACA 6 and 7 is obvious (dead or alive upon delivery to hospital), this mitigates the risk of erroneous registration of NACA 7 vs 6.

## Conclusion

This cohort study found that 27% of all HEMS dispatches were to NACA 5 and 6 patients. Twenty-four percent of these were post-ROSC patients. Sixty-three percent of all patients received at least one advanced physician-requiring intervention, and the average number of interventions were consistent the last 10 years. We conclude that the capabilities that a physician-staffed HEMS resource provide is necessary for a high number of critically ill and injured patients.

### Supplementary information

**Supplementary Material 1.**

**Supplementary Material 2.**

**Supplementary Material 3.**

**Supplementary Material 4.**

## Data Availability

No datasets were generated or analysed during the current study.

## Abbreviations

HEMS: Helicopter emergency medical service
NACA: National Advisory Committee on Aeronautics
ROSC: Return of spontaneous circulation
EMCC: Emergency medicine coordination centre
CPR: Cardiopulmonary resuscitation
