# Supplementary material for: Characteristics of the most severely ill and injured patients in a Norwegian helicopter emergency medical service: a retrospective cohort study
Source: BMC Emerg Med. 2024 Mar 2;24:35. doi: 10.1186/s12873-024-00954-7 (PMC10908032; doi:10.1186/s12873-024-00954-7)
Supplement: Supplementary file 2 — Supplementary Material 2. [file 12873_2024_954_MOESM2_ESM.pdf]

Supplementary file 1. Data tables presenting all intervention entries according to diagnostic group, ROSC group and non-CPR group. ROSC indicates return of spontaneous circulation; CPR, cardiopulmonary resuscitation.

**Total population**	**Cardiac**	**Trauma**	**Vascular**	**Respiratory**	**Other**	**Neurologic**	**Infection**	**Gastric**	**Neonate**	**Obsteric**	**Total**
**Patients (n =)**	**910**	**464**	**101**	**288**	**267**	**382**	**78**	**54**	**36**	**18**	**2598**
NACA 5 (n =)	382	288	78	230	214	289	67	47	27	18	1640
NACA 6 (n =)	528	176	23	58	53	93	11	7	9	0	958
**Pharmacologic intervention**											
Analgetics	350	169	47	93	62	125	20	16	11	-	893
Anesthesia	219	169	11	97	68	189	29	17	7	1	807
Vasoactive medication	440	70	33	100	72	100	41	24	5	1	886
Trombolysis	53	-	-	1	1	3	-	-	-	-	58
Other medications	391	187	41	142	110	185	34	23	16	11	1,140
**Respiratory intervention**											
Intubation/trach	391	8	7	100	63	343	29	18	18	1	978
O2	736	358	63	230	192	289	61	43	28	4	2,004
Ventilator	204	120	6	104	50	130	30	18	19	1	682
Assisted ventilation	437	155	8	146	68	135	21	3	28	-	1,001
Thoracic drainage	4	21	-	9	1	1	1	1	3	-	41
**Circulatory intervention**											
Blood transfusion	8	41	19	1	16	2	7	16	-	2	112
Fluid infusion	591	288	61	149	155	261	66	40	28	6	1,645
Central venous catheter	31	16	2	26	15	8	18	9	3	-	128
Intraosseous access	34	8	-	2	3	-	2	-	-	-	49
Arterial line	221	90	46	84	75	120	40	30	-	2	708
Ultrasound	142	28	15	18	15	5	4	-	-	3	230
**Trauma specific intervention**											
Cervical collar	1	86	-	-	5	7	-	1	-	-	100
Spinal immobilization	5	112	-	-	8	3	1	-	-	-	129
Femur traction device	1	24	-	-	2	3	-	1	-	-	30
**Other intervention**											
Gastric tube	241	102	4	53	47	119	18	12	19	1	616
Urinary cathether	3	4	-	4	1	3	4	3	-	-	22
Incubator	4	-	-	3	4	1	-	-	32	-	44
**Cardiac specific intervention**											
External pacemaker	9	-	1	-	-	-	-	-	-	-	10

Dash (-) indicates 0 entries

Table S1. Interventions in total population, across diagnostic groups.

ROSC group	Cardiac	Trauma	Vascular	Respiratory	Other	Neurologic	Infection	Gastric	Neonate	Obsteric	Total
**Patients (n =)**	**509**	**59**	**2**	**12**	**17**	**15**	**1**	**-**	**4**	**-**	**619**
NACA 5 (n =)	64	2	-	2	7	6	-	-	1	-	82
NACA 6 (n =)	445	57	2	10	10	9	1	-	3	-	537
**Pharmacologic intervention**											
Analgetics	171	12	2	6	8	7	1	-	2	-	209
Anesthesia	177	14	2	7	8	14	1	-	1	-	224
Vasoactive medication	310	15	-	4	8	5	1	-	2	-	345
Trombolysis	21	-	-	-	-	-	-	-	-	-	21
Other medications	190	27	1	5	4	10	-	-	4	-	241
**Respiratory intervention**											
Intubation/trach	358	35	-	9	11	12	1	-	4	-	430
O2	464	55	2	12	15	14	1	-	4	-	567
Ventilator	179	16	-	7	6	8	1	-	4	-	221
Assisted ventilation	371	47	2	9	9	11	1	-	3	-	453
Thoracic drainage	2	2	-	-	1	-	-	-	-	-	5
**Circulatory intervention**											
Blood transfusion	5	1	1	-	2	-	-	-	-	-	9
Fluid infusion	398	36	2	11	14	14	1	-	4	-	480
Central venous catheter	20	1	-	2	2	1	1	-	-	-	27
Intraosseous access	31	4	-	-	-	-	-	-	-	-	35
Arterial line	133	8	1	3	4	8	1	-	-	-	158
Ultrasound	101	4	-	1	4	-	-	-	-	-	110
**Trauma specific intervention**											
Cervical collar	1	6	-	-	2	-	-	-	-	-	9
Spinal immobilization	3	7	-	-	4	-	-	-	-	-	14
Femur traction device	1	1	-	-	-	-	-	-	-	-	2
**Other intervention**											
Gastric tube	224	23	-	8	10	10	1	-	4	-	280
Urinary cathether	1	-	-	-	1	-	-	-	-	-	2
Incubator	3	-	-	1	2	-	-	-	2	-	8
**Cardiac specific intervention**											
External pacemaker	3	-	-	-	-	-	-	-	-	-	3

Dash (-) indicates 0 entries

Table S2. Interventions in ROSC group, across diagnostic groups.

Non-CPR group	Cardiac	Trauma	Vascular	Respiratory	Other	Neurologic	Infection	Gastric	Neonate	Obsteric	Total
**Patients (n =)**	**401**	**405**	**99**	**276**	**250**	**367**	**77**	**54**	**32**	**18**	**1979**
NACA 5 (n =)	318	286	78	228	207	283	67	47	26	18	1558
NACA 6 (n =)	83	119	21	48	43	84	10	7	6	0	421
**Pharmacologic intervention**											
Analgetics	179	157	45	87	54	118	19	16	9	-	684
Anesthesia	42	155	9	90	60	36	28	17	6	1	583
Vasoactive medication	130	55	33	96	64	234	40	24	3	1	541
Trombolysis	32	-	3	1	1	-	-	-	-	-	37
Other medications	201	160	40	137	106	175	34	23	12	11	899
**Respiratory intervention**											
Intubation/trach	33	131	7	91	52	173	28	18	14	1	548
O2	272	303	61	218	177	275	60	43	24	4	1,437
Ventilator	25	104	6	97	44	122	29	18	15	1	461
Assisted ventilation	66	108	6	137	59	124	20	3	25	-	548
Thoracic drainage	2	19	1	9	-	-	1	1	3	-	36
**Circulatory intervention**											
Blood transfusion	3	40	20	1	14	-	7	16	-	2	103
Fluid infusion	193	252	59	138	141	247	65	40	24	6	1,165
Central venous catheter	11	15	2	24	13	7	17	9	3	-	101
Intraosseous access	3	4	-	2	3	-	2	-	-	-	14
Arterial line	88	82	45	81	71	112	39	30	-	2	550
Ultrasound	41	24	19	17	11	1	4	-	-	3	120
**Trauma specific intervention**											
Cervical collar	-	80	-	-	3	7	-	1	-	-	91
Spinal immobilization	2	105	-	-	4	3	1	-	-	-	115
Femur traction device	-	23	1	-	2	1	-	1	-	-	28
**Other intervention**											
Gastric tube	17	79	4	45	37	109	17	12	15	1	336
Urinary cathether	2	4	-	4	-	3	4	3	-	-	20
Incubator	1	-	-	2	2	1	-	-	30	-	36
**Cardiac specific intervention**											
External pacemaker	6	-	1	-	-	-	-	-	-	-	7

Dash (-) indicates 0 entries

Table S3. Interventions in non-CPR group, across diagnostic groups.
