# Supplementary material for: Characteristics of the most severely ill and injured patients in a Norwegian helicopter emergency medical service: a retrospective cohort study
Source: BMC Emerg Med. 2024 Mar 2;24:35. doi: 10.1186/s12873-024-00954-7 (PMC10908032; doi:10.1186/s12873-024-00954-7)
Supplement: Supplementary file 3 — Supplementary Material 3. [file 12873_2024_954_MOESM3_ESM.pdf]

Supplementary file 2. Data table presenting the distribution of advanced interventions across primary and secondary missions.

**Advanced interventions across missions characteristic**	**Primary mission**		**Secondary mission**		**Total**	
	**n =**	**%**	**n =**	**%**	**n =**	**%**
**Number of patients:**	1916	74%	680	26%	2598	100%
Intubation/trach	679	26%	299	12%	978	38%
Vasoactive medication	539	21%	347	13%	886	34%
Anesthesia	525	20%	282	11%	807	31%
Arterial line	320	12%	388	15%	708	27%
Ventilator	372	14%	310	12%	682	26%
Ultrasound	201	8%	29	1%	230	9%
Central venous catheter	12	0%	116	4%	128	5%
Blood transfusion	45	2%	67	3%	112	4%
Incubator	5	0%	39	2%	44	2%
Thoracic drainage	20	1%	21	1%	41	2%

All percentages calculated from total population (n = 2598)

Table S4. Distribution of advanced interventions across primary and secondary missions.
