# Supplementary material for: Characteristics of the most severely ill and injured patients in a Norwegian helicopter emergency medical service: a retrospective cohort study
Source: BMC Emerg Med. 2024 Mar 2;24:35. doi: 10.1186/s12873-024-00954-7 (PMC10908032; doi:10.1186/s12873-024-00954-7)
Supplement: Supplementary file 4 — Supplementary Material 4. [file 12873_2024_954_MOESM4_ESM.pdf]

Supplementary file 3. Thrombolytic treatment protocol of pre-hospital services in central Norway

Background information

Thrombolytic treatment in ST-elevated myocardial infarction is provided in the pre-hospital setting or in local hospitals. If the time until primary percutaneous coronary intervention (PCI) can be performed exceeds 120 minutes – thrombolytic treatment may be indicated. The STEMI diagnosis must be set and confirmed by a hospital physician. EKGs and patient history will be sent electronically by emergency medical services.

STEMI patients with symptom onset within 12 hours should be handled without any delay and be considered for PCI if treatment can be given within 120 minutes after diagnostic EKG. If time to PCI exceeds these limits, thrombolytic treatment is indicated. The patient will be transported to a PCI centre as soon as the patient's condition allows it.

The destination and treatment given is decided by the hospital physician that receives the EKG and patient history. Transport by helicopter is often warranted. Non ST-elevated myocardial infarction patients is usually treated in the local hospital – although this is a clinical decision made by physician on scene or hospital physician[1].

1. 017 ESC Guidelines for the management of acute myocardial infarction in patients presenting with ST-segment elevation: The Task Force for the management of acute myocardial infarction in patients presenting with ST-segment elevation of the European Society of Cardiology (ESC). Eur Heart J. 2018;39:1991–1991.

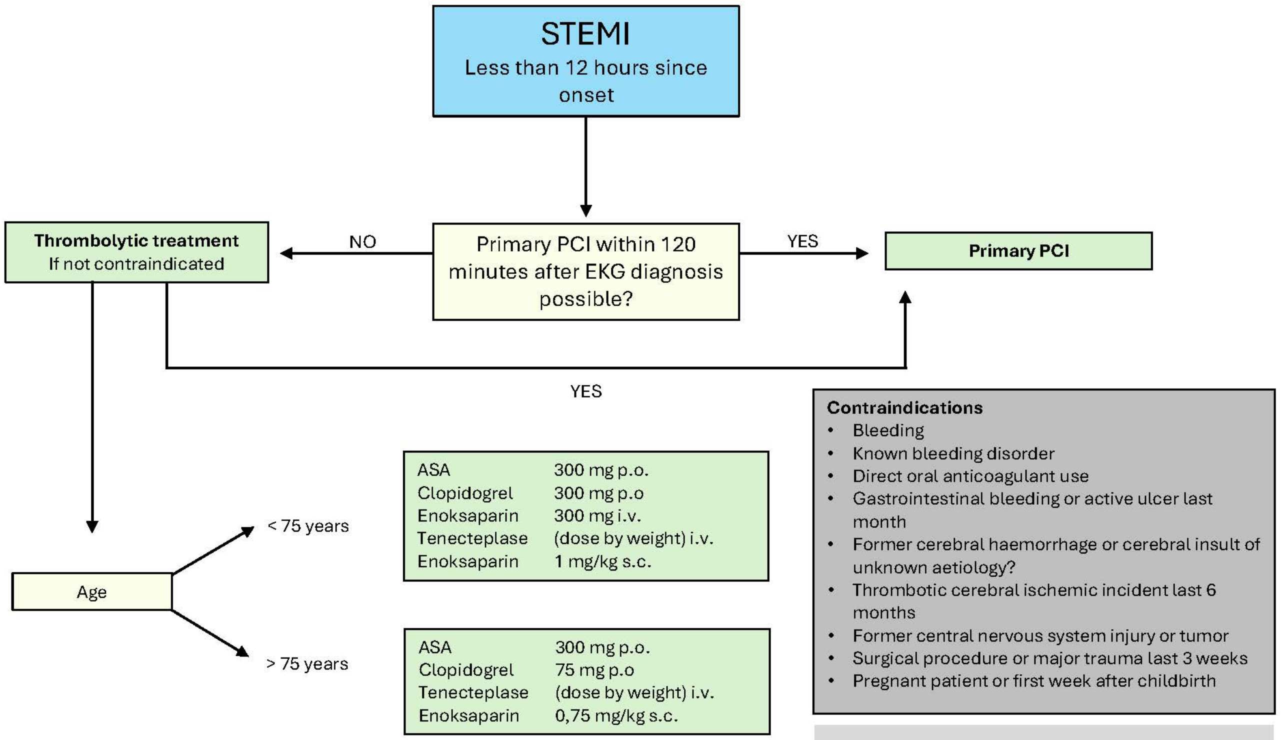

Tenecteplase dose chart

**Patient weight**	**mg**	**U**
< 60 kg	30	6000
60 - 69 kg	30	7000
70 - 79 kg	40	8000
80 - 89 kg	45	9000
> 90 kg	50	10000

Source: Protocol nr. 11485,
Thrombolytic treatment protocol for ST-elevated myocardial infarction (STEMI)
Helse midt pre-hospital guidelines (FRAM)

Figure S1 Flow chart describing thrombolytic treatment protocol. STEMI indicates ST-elevated myocardial infarction.
